# Review on Treatment Planning Systems for Cervix Brachytherapy (Interventional Radiotherapy): Some Desirable and Convenient Practical Aspects to Be Implemented from Radiation Oncologist and Medical Physics Perspectives

**DOI:** 10.3390/cancers14143467

**Published:** 2022-07-17

**Authors:** Antonio Otal, Francisco Celada, Jose Chimeno, Javier Vijande, Santiago Pellejero, Maria-Jose Perez-Calatayud, Elena Villafranca, Naiara Fuentemilla, Francisco Blazquez, Silvia Rodriguez, Jose Perez-Calatayud

**Affiliations:** 1 Medical Physics Department, Hospital Universitari Arnau de Vilanova, 25198 Lleida, Spain; 2 Unidad Mixta de Investigación en Radiofísica e Instrumentación Nuclear en Medicina (IRIMED), Instituto de Investigación Sanitaria La Fe (IIS-La Fe), Universitat de Valencia (UV), 46010 Valencia, Spain; javier.vijande@uv.es (J.V.); joseperezcalatayud@gmail.com (J.P.-C.); 3 Radiotherapy Department, La Fe Hospital, 46026 Valencia, Spain; pacocelada@hotmail.com (F.C.); mariajose.perezcalatayud@gmail.com (M.-J.P.-C.); 4 Medical Physics Department, Hospital San Juan, 03550 Alicante, Spain; jochiher@gmail.com; 5 Department of Atomic, Molecular and Nuclear Physics, University of Valencia, 46010 Valencia, Spain; 6 Instituto de Física Corpuscular, IFIC (UV-CSIC), 46010 Valencia, Spain; 7 Radiation Oncology Department, Hospital Universitario de Navarra, 31008 Navarre, Spain; santiago.pellejero.pellejero@navarra.es (S.P.); elena.villafranca.iturre@navarra.es (E.V.); naiara.fuentemilla.urio@navarra.es (N.F.); 8 Radiotherapy Department, Hospital Clínica Benidorm, 03501 Alicante, Spain; fblazquezm@gmail.com (F.B.); atosrodriguez@gmail.com (S.R.)

**Keywords:** cervix, treatment planning systems, interstitial applicators, magnetic resonance

## Abstract

**Simple Summary:**

There are no brachytherapy treatment planning systems (TPS) exclusively for the treatment of cervical tumours, so general-purpose TPSs are used. However, these treatments have some particular features concerning the treatment of other pathologies, especially in the case of exclusive use of MRI as an imaging modality and the presence of gynaecological applicators in combination with an interstitial part. That is why it is essential to review the latest versions of commercial TPSs to find the potential features to improve with the help of a group of experimented medical physicists and radiation oncologists. Furthermore, after reviewing the recent literature for advances applicable to cervical brachytherapy and through his own clinical experience, possible improvements are proposed to software providers for the development of new tools.

**Abstract:**

Intracavitary brachytherapy (BT, Interventional Radiotherapy, IRT), plays an essential role in the curative intent of locally advanced cervical cancer, for which the conventional approach involves external beam radiotherapy with concurrent chemotherapy followed by BT. This work aims to review the different methodologies used by commercially available treatment planning systems (TPSs) in exclusive magnetic resonance imaging-based (MRI) cervix BT with interstitial component treatments. Practical aspects and improvements to be implemented into the TPSs are discussed. This review is based on the clinical expertise of a group of radiation oncologists and medical physicists and on interactive demos provided by the software manufacturers. The TPS versions considered include all the new tools currently in development for future commercial releases. The specialists from the supplier companies were asked to propose solutions to some of the challenges often encountered in a clinical environment through a questionnaire. The results include not only such answers but also comments by the authors that, in their opinion, could help solve the challenges covered in these questions. This study summarizes the possibilities offered nowadays by commercial TPSs, highlighting the absence of some useful tools that would notably improve the planning of MR-based interstitial component cervix brachytherapy.

## 1. Introduction

In the last few decades, brachytherapy (BT, Interventional Radiotherapy, IRT) has been one of the cornerstones for the treatment of gynaecological lesions, being one of the most widely used, and successful, therapeutic techniques to be considered in these cases. Within gynaecological BT, cervix treatments have experienced the greatest development in recent years. Numerous authors have reported that intracavitary BT plays an essential role in the curative intent of locally advanced cervical cancer management, for which the conventional approach involves external beam radiotherapy (EBRT) with concurrent chemotherapy, followed by BT. Excellent local control rates are achieved with this scheme, as it has been demonstrated by different studies on the curative intent of locally advanced cervical cancer [1,2,3].

In the past decades, the development of new EBRT techniques, such as Intensity Modulated Radiotherapy (IMRT), Volumetric Modulated Arc Therapy (VMAT), Stereotactic Body Radiotherapy (SBRT), or Proton therapy (PT), have broadened the choices of a treatment scheme. In fact, the data of BT use in cervical cancer in the United States (USA) published by Han et al. [4] revealed a trend towards exclusive EBRT treatments, with a decrease in cause-specific survival and overall survival when BT was not employed. In 2013, Tanderup et al. [5] stated that, based on the data collected, exclusive EBRT treatments were not an option when the maximum survival was sought. In the same line, a recent joint publication of the Society of Gynaecologic Oncology (SGO) and the American Brachytherapy Society (ABS) concluded that “conformal external beam therapies such as IMRT or SBRT should not be used as alternatives to brachytherapy in patients undergoing primary curative-intent radiation therapy for cervical cancer” [6]. The National Comprehensive Cancer Network (NCCN) [7] also indicated that, with respect to cervical cancer, SBRT is not considered as an appropriate routine alternative to brachytherapy.

The gynaecological working group of the GEC ESTRO (GYN GEC-ESTRO), formed in the year 2000, is committed to move from 2D BT based on A points to 3D BT based on images, incorporating magnetic resonance imaging (MRI) in the BT planning of the cervix. A new concept of volume definition is proposed, taking into account the topography of the primary tumour at diagnosis and after response to radio-chemotherapy, as well as dosimetric aspects. GYN GEC-ESTRO published its first two recommendations in 2005 and 2006 [8,9], its nomenclature being universally adopted in 2006 [10]. The concept of image-guided adaptive BT (IGABT) thus arises, also called 4D BT, in which the CTV volumes are appropriate for the evolutionary response over time of each BT treatment (changes adjacent to the applicator due to tumour regression, oedema, or changes in organs at risk (OAR)) [11]. The results published at the time were scarce, and more scientific evidence was needed to support, with clinical and toxicity data, the safety of this novel and completely different way of planning the BT component when treating cervical carcinoma. To this end, the first large-scale prospective study on MRI-based cervix BT, EMBRACE I (International study on MRI-guided BRAchytherapy in Cervical cancer), was designed. The EMBRACE Collaborative Group published in 2010 and 2011 recommendations III and IV for the reconstruction of the applicators and image in IGABT [12,13]. Throughout these years, the number of publications with conclusive results have increased, both in single-institutional series [14,15,16,17,18], a multicentre retrospective study RetroEMBRACE (retrospective study of patients treated with IGABT based on CT or MRI before the beginning of EMBRACE [19]), and, recently, the definitive comprehensive data from EMBRACE I [20], with the consolidation of MRI-based IGABT for the treatment of cervical carcinoma. The recommendations of the GEC ESTRO (I–IV) have been used as a conceptual basis worldwide to introduce the IGABT, now being integrated into the current ICRU 89 [21].

Once adopting the use of MRI, it has been evidenced that the interstitial component added to the intracavitary one achieves a higher dose conformity and organs at risk sparing. It also allows the use of different combined applicators options depending on the manufacturer [22,23]. Prescription and reporting have also evolved from 2D, exclusively based on dose points (e.g., A points), to be focused nowadays on metrics based on Dose Volume Histograms (DVH) and the specific anatomy of the patient, as obtained from 3D images. GEC-ESTRO publications [8] introduced DVH metrics (D90, D2cc) for both prescription doses and constrains to OARs [24]. The most updated version of the GEC-ESTRO recommendations has been included in an ongoing prospective observational analysis, EMBRACE II [25]. The objective of that study is, on the one hand, the control of local, lymph node and systemic disease with decreased toxicity and, on the other hand, the definition of patients at risk (including translational research), effect of techniques of external radiotherapy, such as IMRT/VMAT, increased lymph node dose with integrated boost, and increased use of interstitial BT in IGABT.

Radical locally advanced cervical cancer treatment comprises pelvic EBRT with concomitant chemotherapy, following BT. The EBRT dose is usually 45–50 Gy in 1.8–2 Gy fractions. Lymph nodes, parametria, or pelvic sidewall boosts up to 55–64 Gy are used, depending on the clinical case and the experience of each individual centre [18,19]. Although HDR-BT schemes of 5.5–6 Gy × 5 fractions are recommended in some guidelines, the “nominal” 28 Gy in 4 fractions scheme is well-established thanks to the EMBRACE trials [26]. Usually, these four HDR-BT fractions are administered in two applications in 1–2 weeks, at the end of EBRT or intercalated between the last EBRT fractions. Overall treatment time, an independent prognostic factor for local control, should remain below 50–55 days [27].

As recommended by ICRU 89, the difference in the biological effects of the EBRT and BT fractions makes it necessary to define a parameter to evaluate them globally. The previously referenced publications make use of the dose equivalent of 2 Gy per fraction (EQD2), using the linear-quadratic model with α/β = 10 Gy for tumour effects and α/β = 3 Gy for late normal tissue damage, assuming a half-time repair time of 1.5 h. Therefore, following the ICRU 89 recommendations, EQD2 is the chosen option to sum the absorbed dose to report dose for planning aims, prescriptions, and delivered dose.

Clinical departments use external tools, such as the GEC-ESTRO spreadsheet, to calculate the resulted EQD2 for each patient. None of the current brachytherapy treatment planning systems (TPS) consider the fully CTV-OAR dose balance during the optimization, becoming a user-dependent procedure mostly based on the physicist experience.

Ideally, the reconstruction of the applicator geometry and its matching with the patient anatomy should be done in the same image study in which the radiation oncologist defines the CTV and OARs volumes in T2 weighted MRI. However, there is an important limitation to that technique when using dummies to identify the potential source trajectory for the interstitial component using plastic catheters. Namely, the void signal produced by the air-filled interior of the applicators makes it difficult to set the catheter tips. Therefore, the use of other image modalities is sometimes beneficial in the reconstruction process.

Therefore, the aim of this work is to review the different methodologies used by all commercially available TPSs to solve the main planning issues in an exclusive-MRI-based cervix BT with interstitial component treatment. Moreover, some desirable or convenient practical aspects to be implemented in future TPS versions are outlined from both radiation oncologist and medical physicist perspectives.

## 2. Materials and Methods

The authors of this review are radiation oncologists and medical physicists with extensive experience in MRI-based cervix HDR brachytherapy treatments with an interstitial component, representing the Spanish centres with the largest number of implants performed. The three TPSs analysed were Brachyvision v16.0 from Varian (Varian Medical Systems, Palo Alto, CA, USA), Oncentra v4.6.2 from Elekta (Elekta AB, Stockholm, Sweden), and Sagiplan v2.2 from BEBIG (Eckert & Ziegler BEBIG GmbH, Berlin, Germany). Most of the authors are users of Oncentra Brachy, with just one user of Brachyvision; there are no Sagiplan users.

The reviews of the TPSs were done based on the authors’ clinical experience and interactive demos provided by the software manufacturers. In these demos, the TPS version tested was the latest one commercially available, including also all the new tools currently developed to be included in future releases. After an introduction to most of the utilities and tools of the planning systems, the specialist from the vendor company (henceforth named specialist) was asked to answer some of the issues that are routinely faced in a clinical environment and that will be briefly described below. In order to follow a systematic approach on the evaluation of each TPS capabilities, demos were carried based on the questionnaire summarized in Table 1. Specialists were also asked about external software and devices commercially available or under development that could shed some light on these problems.

The work plan has been complemented by a review of the literature on this specific topic. The bibliography review methodology was based on a keyword search in the PubMed database of publications from the last ten years. Such keywords include MRI, cervical cancer, HDR brachytherapy, catheter reconstruction, dose accumulation, etc. On the other hand, more specific keywords, such as deep learning, electromagnetic tracking, auto-segmentation, and synthetic-CT, were also included. The study aims to highlight the general shortcomings of the TPSs and the possible improvements that could be made to them in the opinion of a group of experienced users. However, it is not the authors’ intention to make comparisons between them, much less to recommend a ranking.

### 2.1. Q1—Tools for Commissioning and QA

Aside from the verification of the applicator’s geometry, the medical physicist must check the TPS calculation of the dose (based on TG43 and also TG186 when possible), the applicator’s geometry within the digital libraries, and all the tools involved in a treatment planning. Digital libraries reproduce the geometry of the applicators. However, the real path of the source could differ from the symmetric axis of the applicator, which is usually the path introduced by the manufacturer in the corresponding libraries [12]. This effect appears predominantly on curved applicators (e.g., ring). Medical physicists should analyse the possible discrepancies during the commissioning period and correct the source path introduced in the digital libraries when the TPS allows it. MBDCAs should be also commissioned. The AAPM/ESTRO/ABG working group on MBDCA in brachytherapy (WG-DCAB) has developed and validated using several test cases for clinical users to perform a standardized commissioning process, including a generic GYN shielded applicator. These tests have been implemented in the two TPSs that include this possibility and shared via the Joint AAPM/IROC-Houston Brachytherapy Source Registry [28]. Vendor-specific manuals have also been shared to guide the physicists.

TPSs should be compatible with these generic models and facilitate the MBDCA commissioning by implementing the tools necessary to perform an in-depth comparison of the 3D dose distributions. Finally, TPSs must easily perform the quality control tests suggested in the guidelines [29,30,31].

### 2.2. Q2—Image Registration and Utilities to Optimize Information from Previous Implants

GYN IGABT image registration would be a fundamental tool to use in different phases of the planning process, such as in applicator reconstruction, volumes definition or propagation, dose accumulation in multifraction BT, or dose accumulation of EBRT + BT.

In the case of applicator reconstruction or when transferring the target volume contours of a first MRI implant to the scans used for a second implant, for images with the applicator in place, a rigid registration of the applicator is recommended [21,32]. Rigid registration can also be used when a CT study is performed prior to the administration of the second fractions of each implant, allowing verification of the position and geometry of the OARs at the time of administration of these fractions. This will offer the possibility to rectify the dosimetry if necessary.

If the brachytherapy treatment consists of more than one implant, it would be very useful to include the dose distributions of previous implants in the optimization.

Since the brachytherapy applicator completely deforms the anatomy compared to the EBRT images, rigid registration has been questioned as a valuable method. Deformable registration could, in principle, combine the dose from each tissue voxel in the EBRT fractions with the corresponding one for each BT fraction.

### 2.3. Q3—MRI Contouring:Removing the Endocavitary Component

A significant issue for cervix segmentation is the presence of the applicator. Such an applicator causes deformation of the surrounding tissues, hindering the correct segmentation [33]. Additionally, the high dose gradient in the vicinity of the applicator may impact the accuracy of the surrounding tissues’ DVH dosimetric parameters [34,35]. These issues will increase the dose uncertainty. Therefore, it is necessary to develop techniques to remove the applicator from the image, not only for accurate tumour segmentation but also for a more accurate DVH evaluation.

### 2.4. Q4—Catheter Reconstruction: Endocavitary Component Library

Determining the source path and the most distal dwell position, together with the matching of the anatomical geometries, are the catheter reconstruction objectives [13]. The CT dummy design that allows a direct reconstruction (DR) of the brachytherapy source channels is straightforward, and all vendors include them in their product catalogues. By contrast, applicator reconstruction is more challenging when MRI is used and even more so in T2-weighted sequences. The materials visible in MRI are usually liquids, and this limits the construction diameter of the dummy. Some solutions, however, are available for the endocavitary part [36].

An alternative to this modality of reconstruction is the use of applicator libraries containing accurate 3D models. The corresponding applicator can be selected and then displaced and rotated until it matches the image using reference points located both in the image and the model. Thereby, the source path and the most distal dwell position are clearly defined. This method is only valid for rigid applicators and, therefore, excludes the interstitial part.

### 2.5. Q5—Needle Reconstruction: Interstitial Component

As discussed in Q4, the use of applicator libraries is only helpful in the case of rigid applicators and therefore the use of applicator libraries is, in principle, not possible for the interstitial part. Thus, direct reconstruction is the only way to make such reconstruction. In contrast to the case of needles on CT images, identifying needle trajectories and needle tips is still an open issue in MRI.

### 2.6. Q6—Interpolated Images

In the case of the DR modality, the determination of the most distal dwell position involves an extra challenge due to the finite slice thickness. Even though a slice thickness lower or equal to 3 mm is suitable for MRI images, the acquisition protocols of a given institution may not offer such a possibility while still needing to achieve millimetric precision on the reconstruction. This precision goal is especially important for the tip position. A possible solution is to add reconstructed images between two slice thicknesses, thus reducing the uncertainty.

### 2.7. Q7—Use of EQD2 in the Optimization Process

Objective and tolerance doses on locally advanced cervix cancer are expressed in EQD2 and not in physical dose units. This accounts for the fact that two treatment modalities with different fractionations and biological effectiveness are intended to be combined (EBRT + BT).

Independently of the method, optimization is based on an iterative process in which the medical physicist and radiation oncologist verifies whether all objective and tolerance doses (in EQD2) are fulfilled. At the time of publishing this review, these iterations are done using an external spreadsheet.

### 2.8. Q8—EQD2 Combination with EBRT: Optimal and Mandatory Constraints

As previously mentioned, the most used parameter for combining the biological effect of EBRT and BT fractions is EQD2. Therefore, mandatory and optimal constraints should be expressed using that quantity. If TPSs can combine the doses of the different fractions in an agile way, an excess or lack of dose can be detected in the EBRT phase and compensated during the BT phase. In addition, for the definition of the intermediate-risk clinical target volume (CTV-IR), it is necessary to transfer the pre-EBRT GTV to the MRI of the BT treatment (ICRU 89) [21].

### 2.9. Q9—Dwell-Time Locking

The most recent protocols (EMBRACE II and ICRU 89) suggest de-escalating vaginal doses (vaginal TRAK representing less than 30% of total) and controlling the contribution from the interstitial component (less than 20–30%) [21,25]. This question was intended to identify the tools that the TPSs have in order to facilitate the control of the contribution of the different components. As an example, dwell-time locking allows blocking a particular component (or just several needles), so it cannot be modified by the inverse optimization algorithm, manual optimization, or renormalization tools. As a result, the intracavitary and interstitial components could be graphically optimized separately.

### 2.10. Q10—Optimization Methods: Implementation of D_90_ and D_2cc_

Once the catheters are reconstructed, the dwell times must be chosen to fulfil the prescribed dose to the target volumes. To this end, several optimization methods have been used. This question was included to get details on the different optimization methods available for each TPS and their limitations. Specifically, the specialists were questioned about the inclusion of inverse-optimization algorithms and the possibility to aim the optimization towards the dosimetric metrics suggested to be reported (D_90_, D_2cc_, etc.). Moreover, the capability of the optimizers to control the dwell time gradient/homogeneity and the weight of each component (intracavitary or interstitial) was also assessed.

### 2.11. Q11—DVH Resolution

Following the advice of ICRU 89, the EMBRACE group decided to abandon the dose report based on minimum (D_100_) and maximum doses. Instead, more robust dosimetric metrics were gathered: D_98_, D_90_, D_2cc_, and D_0.1cc_, among others. The control of the DVH resolution limitations is extremely important in the case of the OAR for which D_2cc_ and D_0.1cc_ are suggested to be reported. This question was aimed to identify the strategies of the different TPSs to be able to identify the doses deposited for volumes up to 0.1 cm.

### 2.12. Q12—D_2cc_ Location

A D_2cc_ value above a certain threshold is the cause of toxicities in OARs. The ratio between the D_2cc_ and ICRU bladder point dose is correlated with the development of urinary morbidity [37,38]. Mazeron et al. also found an increased likelihood of rectal bleeding when the rectal D_2cc_ was greater than 70 Gy. If the position of D_2cc_ is known, it would be possible to take this information into account during the optimization process (i.e., manual fine tuning).

### 2.13. Q13—Model-Based Dose Calculation Algorithms (MBDCA)

Introduction of MBDCAs capable of accounting for tissue and applicator heterogeneities in brachytherapy has been a major development in brachytherapy treatment planning in recent years. Their emergence and the protocols for early adopters was addressed by AAPM, ESTRO, ABS, and ABG Task-Group 186 (TG186) [39]. TG186 emphasizes that although prescriptions based on the TG-43 dose calculation formalism must remain in effect, they should be compared against MBDCA planning systems to understand its possible shortcomings and limitations. For this reason, the availability of MBDCAs was checked for each TPS.

## 3. Results

After the specialists presented the demonstrations, they answered the questions raised by the authors. Their answers to the questionnaire are summarized in this section following the scheme outlined in the Materials and Methods section, together with the authors’ comments.

### 3.1. A1—Tools for Commissioning and QA

Only one TPS allows the modification of the source path within the applicator’s library. It is essential for curved applicators, such as rings or ovoid ones, where the source usually moves close to the applicator wall and far from the axis of symmetry.

Out of the three TPSs analysed, only two of them include the possibility of performing MBDCA calculations. None of the test cases available at the time of writing this manuscript are clinical cases, with WG-DCAB currently developing specific test cases for GYN. However, Test Case 4, although it does not fully resemble a clinical situation, incorporates a shielded generic GYN application, and therefore it is the most interesting one for the GYN clinical user to commission his/her TPS. None of the TPSs analysed incorporate a complete set of tools to perform that commissioning process, requiring the clinical user to use external tools, such as BrachyGuide [40] or AMIGO [41].

Regarding the commissioning of the dose calculation by TG43, the authors believe that the verification procedure is not as operational as it should be. The dose that the TPS returns for specific points will be compared with the consensus along–away tables using the TG43 protocol functions for a given source model. However, these points can only be entered via the keyboard and one at a time. A more agile and less error-prone method is desirable.

### 3.2. A2—Image Registration and Utilities to Optimize Information from Previous Treatments

All three TPSs allow rigid registration, although only one includes the possibility of deformable image registration.

In theory, deformable registration would match and sum each voxel dose combining each fraction of EBRT with the corresponding contribution of BT. However, none of the available TPSs include the technology required to track the effect on biologically meaningful structures within target volumes and OARs without using external compatible software. For this reason, GEC-ESTRO recommends assuming that the organ walls adjacent to the brachytherapy applicator and target volumes received the EBRT prescribed dose. Such a conservative approach, already widely used in clinical practice, has provided reliable results for both target and non-mobile OARs, despite being a rather important assumption. In addition, following one of the EMBRACE II recommendations for the planning of EBRT, a control region around the high-risk clinical target volume (CTV-HR) of 10 mm thickness could be generated to establish a dose homogeneity requirement in order to avoid hotspots in the OARs close to the CTV-HR, which are likely also to receive a considerable BT dose. Special attention should be paid in EBRT cases with multiple PTVs and doses due to the largest OAR D2cc dose.

### 3.3. A3—MRI Contouring: Removing the Endocavitary Component

None of the TPSs allow removing the ring or ovoids from the MRI. In the case of using applicator libraries for reconstruction, information on the position of the applicator together with its composition could help resolve this issue in future versions of the TPSs.

### 3.4. A4—Catheter Reconstruction: Endocavitary Component Library

All TPSs analysed have access to intracavitary applicator virtual libraries for catheter reconstruction. As previously discussed, it is useful to facilitate and reduce the uncertainty in the reconstruction phase. The DR is an alternative to this type of reconstruction and the only solution for non-rigid applicators, especially for the commercially available interstitial part. However, taking the example of reconstruction of a ring applicator that includes a tandem, both parts should be treated as two different applicator library elements. Nevertheless, the tandem is joined to the ring, so they are not strictly independent and may offer additional geometrical constraints that might be used to improve its reconstruction. It would be desirable to further develop this idea.

All specialists mention the intention of improving the auto-placement algorithm on MRI image sets because they recognized the inherent uncertainty in all cases. TPSs already incorporate such tools, but they are only accurate when used on CT images. Hrinivich et al. [42] have developed a self-reconstruction method of ring and tandem using T2-weighted MRI images with an algorithm based on three-dimensional surface model registration, optimized by maximizing the image intensity gradient normal for the model surface.

### 3.5. A5—Needle Reconstruction: Interstitial Component

In the case of the reconstruction of the interstitial part, TPSs allow reconstructing the needles through the black area left by the plastic needles and the offset between the tip and first dwell position. However, in some cases the black area is not seen clearly enough to perform such reconstruction efficiently.

Along the same lines, as mentioned for the intracavitary part, the needle path is not entirely independent of the rest of the applicator. Richart et al. [43] suggest a method for reconstructing the needles in the Utrecht applicator (Elekta, Veenendaal, The Netherlands) based on the inserted length of each needle as reported by the radiation oncologist at the moment of the insertion. This distance is determined using a ruler engraved on the insertion tool. In other words, the distance between the applicator exit hole and the needle tip is the distance called the free length.

Taking the free length concept, Otal et al. [44] developed a method to include the interstitial component as an element in the applicator library. In such a model, the needles come out from the holes in the ovoid. They have a length equal to the free length and the direction of the cavity in the ovoid. Once the first part is in position, the needles are placed over the black area, making rotations around the exit hole, keeping the exit point on the ovoid invariant.

In parallel to solutions based on applicator libraries, some groups continue to work on direct reconstruction methods. Although needle diameter is a challenge for the development of dummies, there are possible solutions already under development. Shaaer et al. [45] have tested an MRI line marker for the interstitial component. In a subsequent publication, with the help of those markers, the same group performed an automatic catheter segmentation using a convolutional neural network using the U-Net model, making a needle reconstruction after a post-process of the previous segmentation [46].

### 3.6. A6—Interpolated Images

Two out of the three TPSs studied include the probability to access “interpolated slices” between the acquired ones. The third TPS has needle models in its applicator library but just in the case of straight ones. All specialists mentioned the manufacturer’s intention of enhancing the auto-placement algorithm on MRI image sets because they recognized the uncertainty in current TPS versions.

### 3.7. A7—Use of EQD2 in the Optimization Process

Only one of the TPSs has the option to import the dosimetric information of the fractions delivered. In this case, the DVH information is directly added to the BT planning and transformed to EQD2 units based on α/β values introduced by the user. There is no summation of dose distributions and the DVHs are only extracted from the EBRT fractions. It would be more convenient if such an iterative process were integrated into the TPS and incorporated into the optimization phase.

### 3.8. A8—EQD2 Combination with External RT: Optimal and Mandatory Constraints

To obtain the biological effect of the EBRT and BT phases in terms of EQD2, it is necessary to register all BT fraction delivered as accurately as possible [47]. Although all specialists agreed on the importance of this point, current TPSs lack the tools required to optimize the BT fractions considering the previously delivered EBRT fractions.

Swamidas et al. [32] and Kim et al. [48] provide an overview of the current status of image registration for dose accumulation in image-guided gynaecological brachytherapy, including combination with external beam radiotherapy. Both review studies conclude that although deformable image registration algorithms are a promising tool for dose accumulation for EBRT, BT, and multi-fractionated BT, it still requires further research and development before they are ready for clinical application, especially to assess the uncertainties arising from deformable registration.

Current TPSs have limitations when calculating cumulative dose distributions and when deriving a composite DVH from EBRT and BT plans in EQD2. It would be desirable to promote the research and evolution of deformable registration algorithms and adapt them to the complexities of cervical radiotherapy treatment planning.

### 3.9. A9—Dwell-Time Locking

All TPSs includes several tools for modifying the dwell times: manual variation of times, normalization to points or reference lines, general renormalization, and graphical optimization, among others. The ability to lock individual catheters and dwell positions, making the dwell times non-modifiable, was only included in one TPS. This tool is helpful since it facilitates the control of the weight of each component (tandem, ovoids/ring, and interstitial).

### 3.10. A10—Optimization Methods: Implementation of D_90_ and D_2cc_

Inverse optimization based on the objective dosimetry metrics (D_90_, D_2cc_, ...) is offered in all TPSs. The D_2cc_ value extracted from the DVH is also reported. Even though these optimization modules have the potential to offer clinically acceptable dose distributions, none of the TPSs provided the required control of the dose gradient around needles despite having specific parameters to modulate it. Another interesting tool already incorporated in the EBRT inverse optimization algorithms is the possibility of starting from a solution given by the user and using it as an initial state in the optimization process. BT TPSs will benefit from incorporating a similar feature.

### 3.11. A11—DVH Resolution

Only one of the TPS analysed handles this issue properly. In the other cases, it was not clearly specified.

### 3.12. A12—D_2cc_ Location

All the specialist agreed that the location of the D_2cc_ position in the OAR is essential. One of the TPSs includes an option that locates the plane with the maximum value for a particular structure, showing the contribution of each source to that point. The rest do not mention any similar solution.

A numerical value of D_2cc_ as a restriction on the rectum and bladder is not enough to predict subsequent toxicities. It is also essential to know the position in that organ. The particular location of these high dose points may require a re-optimization of the dose distribution. Therefore, the authors argue that it is crucial to include in the TPSs tools that make these high-dose areas visible or, even preferably, to include these positions as input data in future optimization algorithms.

### 3.13. A13—Model-Based Dose Calculation Algorithms (MBDCA)

Due to its clinical relevance, the cervix treatments were among the first to be analysed from the perspective of MBDCA. Retrospective assessments of the relevance of heterogeneities in the case of the conventional Manchester system plans have been performed [49,50,51]. A small impact on the TG-43 calculated dosimetric parameters was reported, observing minor changes in Point A and B doses and the D_2cc_ and D_90_ volumetric parameters. Hot and cold spots of about a 10% difference were observed at particular locations within the imaging volume while attenuation in the titanium applicator walls contributed about 1.3% to these reductions. Hofbauer et al. [52] re-evaluated the treatment plans delivered with plastic tandem and ring applicators and an interstitial technique with 3 to 10 additional needles when necessary. The authors reported a minimal dosimetric impact, with D_90_ and V100 for high-risk CTV reduced by less than 0.5% and D_2cc_ and D_0.1cc_ for organs at risk reduced by less than 2%. Abe et al. [53] evaluated the impact in the rectum D2cc of its gas content in patients treated using different techniques. The authors reported differences with respect to TG-43 in the range 11.9 ± 2.6% (full gas content) to 0.8 ± 2.0% (filled with water-equivalent material).

Therefore, it is clear that, although MBDCA may offer additional insight into the dose deposited, the clinical impact of the differences reported with respect to TG-43-based clinical parameters is negligible for MRI-based cervix brachytherapy.

### 3.14. Summary

In summary, the main points that commercial TPSs would be requested to improve significantly in their planning procedures would be:

- More agile data insertion, making it possible to work with data lists and more advanced tools that help in the commissioning and QA of the TPS.
- Regarding applicator reconstruction, explore the possibilities of automatic reconstruction using, for example, AI-based tools. Furthermore, research on the design of new dummies is needed regarding needle reconstruction and the use of such new tools.
- It would be helpful if the optimization algorithms could handle biological dose equivalents (EQD2) to dispense with external tools.
- Due to the relationship of “hot spots” in OARs with their toxicity, a suitable tool for locating such spots would be desirable.
- Finally, integrating deformable registration algorithms in the TPS would be very helpful.

## 4. Discussion

The main objective of this study is to propose improvements and point out the gaps in the existing TPSs. External software compatible with the TPSs that could help improve the issues outlined here were excluded from the study, including those scripting environments within the TPSs that may allow the user to program personalized tools. As discussed above, this review is based on the clinical expertise of a group of radiation oncologists and medical physicists. Interactive demos provided by the software manufacturers and delivered by specialists from the supplier companies were also used. These specialists were asked to propose solutions to some of the challenges through a questionnaire. Possible limitations of this methodology are the following. It is expert- and specialist-opinion based, therefore personal biases are always a risk. In this case, considering that it is a relatively large group of professionals working in different institutions (different protocols, installations, background, etc.), such an issue is diminished. Another possible limitation is the fact that not all experts have used all TPSs in their clinical practice, something unavoidable considering the realities of clinical practice. The use of questionnaires also might limit the study, since they inherently have selection and sampling biases that might preclude the discovery of unforeseen situations.

In addition, this modest multicentre and multidisciplinary study is fundamentally aimed at pointing out deficiencies and limitations rather than quantifying their consequences.

There is a segmentation software that incorporates more advanced tools for image registration that include utilities for dose summation between different fractions. It would be desirable that such, or similar, tools eventually make their way to the TPSs.

Other tools, this time related to the segmentation of clinical volumes and organs at risk, that would be interesting to incorporate into brachytherapy-specific TPSs would automate such segmentation by means of convolutional neural networks. These are already being incorporated into the clinical workflow in radiotherapy, and there are recent works in the literature explicitly oriented to the cervix case [54,55,56], although CT is the imaging modality used in these works.

An exciting topic in external beam therapy is the generation of synthetic CTs from MRI. The main objective of the generation of such CTs is to segment tumours and organs at risk in the MRI and obtain the electron densities necessary for dose calculation [57]. These synthetic CT scans could be helpful in the case of cervical brachytherapy and are nowadays an unexplored line of research. The user community has historically developed ‘in-house’ tools that attempt to make up for the shortcomings of the TPSs and cannot typically be incorporated into the planner’s workflow. A commonly known example are spreadsheets. A robust scripting environment in a high-level language would help the users in optimizing their work routines and also be a source of inspiration for companies integrating future product development.

In the last years, several research groups have been working on electromagnetic tracking systems [58,59,60,61] to check the trajectory of the brachytherapy source before the treatment delivery. One of the specialists is participating in a project involving such technology. Although its primary purpose is to verify the path followed, it could be used as a self-reconstruction tool in the future. Nowadays, it is not available to users.

Some studies have recently been published investigating the feasibility of deep learning-based algorithms for semi-automated reconstruction of interstitial catheters during MRI-based gynecological HDR [46].

## 5. Conclusions

This review has been performed by radiation oncologists and medical physicists with extensive clinical experience in MRI-based cervix HDR brachytherapy treatments with an interstitial component, representing the Spanish centres performing the largest number of implants. The main goal being reviewing the different methodologies used by all commercially available TPSs to solve the main planning issues for such a technique. Some desirable or convenient practical aspects to be implemented in commercial TPSs are outlined from the perspective of both radiation oncologists and medical physicists. This study also highlights the absence of some useful tools that would notably improve the planning of MR-based cervix brachytherapy with an interstitial component.

## Figures and Tables

**Table 1:** The questionnaire discussed with each TPS manufacturer specialist.

Question	Description
Q1	Tools for commissioning and QA.
Q2	Image registration and utilities to optimize information from previous treatments data.
Q3	MRI contouring. Removing the endocavitary component.
Q4	Catheter reconstruction. Endocavitary component library.
Q5	Needle reconstruction. Interstitial component.
Q6	Interpolated images.
Q7	Use of EQD2 in the optimization process.
Q8	EQD2 combination with external beam radiation therapy. (EBRT). Optimal and mandatory constrains.
Q9	Dwell times locking.
Q10	Optimization methods. Implementation of D90 and D2cc.
Q11	DVH resolution.
Q12	D2cc location.
Q13	Model-based dose calculation algorithms (MBDCA)

The rationale underlying each question is discussed below.
